# Working With Refugees' Health During COVID-19—The Experience of Health- and Social Care Workers in Sweden

**DOI:** 10.3389/fpubh.2022.811974

**Published:** 2022-05-19

**Authors:** Elisabeth Mangrio, Slobodan Zdravkovic, Michael Strange

**Affiliations:** ^1^Department of Care Science, Faculty of Health and Society, Malmö University, Malmö, Sweden; ^2^Malmö Institute for Studies of Migration, Diversity and Welfare (MIM), Malmö University, Malmö, Sweden; ^3^Global Political Studies, Malmö University, Malmö, Sweden

**Keywords:** COVID-19, healthcare, health professionals, migrants, health literacy

## Abstract

**Introduction:**

In Sweden, often seen as one of the most egalitarian countries, the COVID-19 pandemic exposed high levels of health inequality, especially harming people with a refugee background. This is also despite Sweden's image as a refugee-friendly country. In this context, the aim of this paper is to better understand how Swedish health- and social workers have reacted to the health- and social needs of refugees during the pandemic. The Swedish case is particularly interesting because, as seen in the paper, health- and social workers had the task of communicating health guidance to refugees who were sometimes more reliant on information from abroad where the consensus on COVID-19 restrictions ran contrary to the approach recommended by the Swedish public health authority.

**Method:**

The study utilizes a qualitative content analysis of 13 in-depth interviews with health- and social workers in Sweden, active in the care of refugees within different kinds of health- and social care settings.

**Results:**

The analysis showed that healthcare services have remained open during the pandemic but with new precautions at reception areas impacting how refugees access healthcare. As discussed in the article, the shift to digital tools has particularly impacted refugees, worsening already existing barriers to healthcare services faced by those with refugee status. Public health recommendations were poorly designed to the needs of refugees whose living conditions often prevented them from self-isolation and social distancing. Furthermore, Sweden's initially non-restrictive approach to the pandemic instructed health- and social-workers to encourage refugees to take far fewer precautions (e.g., self-isolation, home-schooling, pregnant women to avoid virus hotspots) compared both with European neighbors and the international media typically used by refugees. When Sweden shifted toward a more restrictive approach, health- and social-workers had to revise their guidance in relation to the new recommendations around precautions.

**Conclusion:**

Refugees have faced increased barriers to maintaining their health and wellbeing during the pandemic that exceed those experienced by the rest of the Swedish population. Refugees have, in general, taken precautions in regard to social distancing and followed recommendations but faced challenges with social distancing due to isolation and crowded living. Public health authorities have often failed to acknowledge that individuals use increasingly diverse sources of knowledge when trying to protect their health, and that not everyone has access to the knowledge needed to access healthcare and social systems. At the same time, there is a need to acknowledge that refugees are sometimes a source of expertise that was ignored by the Swedish health and social system during the pandemic. There is a need for urgent efforts to halt the worsening health conditions for this specific group, but also to counter knock-on societal effects and rising health inequity.

## Introduction

The COVID-19 pandemic exposed high levels of health inequality in Sweden—often seen as one of the world's most egalitarian states (1). People living within Stockholm's poorer socioeconomic areas were 3–4 times more likely to get infected by COVID-19 compared to people living within wealthier socioeconomic areas (2), explained by differences in health, susceptibility to infections and crowded living conditions between different socioeconomic areas (2). Health inequality in Sweden has particularly negatively impacted those with a refugee background. For example, refugees already exposed to vulnerable situations regarding social integration in society have suffered societal barriers in regard to healthcare and social services (3) as well as a higher proportion of deaths due to COVID-19 (4). In particular, there has been many more COVID-19 fatalities amongst refugees from low or middle income countries (5). Excess and disproportionate COVID-19 mortality amongst refugees in Sweden has been explained by their exposure to overcrowded housing and living in socially deprived areas, including difficulties in accessing healthcare services and information during the pandemic (4). Prior to the pandemic, refugees have been recognized as a vulnerable population in Swedish society with higher levels of mental ill-health, social isolation, family separations, lack of employment and unstable and crowded living conditions research (6–12). Recently published data on COVID-19 and migration in Europe, shows that migrants living in Sweden, Norway, Spain, and the UK became infected by COVID-19 at a higher rate compared to the rest of the population within these countries (13, 14). Another recently published study, focused on Swedish residents, shows higher COVID-19 death rates amongst refugees from the Middle East and Africa compared to the rest of the population (15), which is partly explained by lower socioeconomic position and poor housing conditions (15).

The health care system in Sweden, as well as globally, has faced challenges in order to maintain its function during the recent and ongoing pandemic, including the lack of sufficient resources for covering health care during the pandemic (16). In recent years, prior to the pandemic, there has been evidence that refugees faced significant challenges seeking healthcare in Sweden (6, 7, 17). These include difficulties with booking appointments, lack of trust in doctors, language issues, and economic barriers (17). When health care access in society is reduced, it is usually first felt by refugees, since they face challenges to prioritize their own health whilst living in an often precarious situation (3). These existing obstacles were worsened due to restrictions intended to control the pandemic, with refugees at particular risk of being excluded from healthcare services due to the shift away from in-person consultations to digital ones (3).

Sweden provides an important case for studying how refugees responded to the pandemic situation and their strategies for countering obstacles. Sweden has for several decades been seen as a generous receiver of refugees even if refugees arrivals have greatly declined in recent years (18). Since 2010, Sweden has provided newly arrived refugees with a settlement programme intended as an introduction to Swedish society and the employment market (19, 20), and to help refugees learn the language, get to know the culture, as well as attain employment. Sweden's welfare system provides healthcare on a comprehensive basis, with a focus on equal health for all people in Sweden (21). Given both Sweden's image as a refugee-friendly country and a proponent of health equity, it is important to better understand the experiences of Swedish health- and social workers in assisting refugees during the pandemic.

Health literacy is central to health promotion and is an important factor in Sweden's treatment of refugees during the pandemic, as will be argued further on (22). Health literacy has been defined as “the cognitive and social skills which determine the motivation and ability of individuals to gain access to, understand and use information in ways which promote and maintain good health” (p. 20) (23). Nutbeam has defined three levels of health literacy, the first being *functional* health literacy, which relates to the basic skills of writing and reading and consists of the level of knowledge and understanding of information about possible health risks and the use of health services (24). The second one is *interactive* health literacy, which refers to more advanced cognitive and social skills that enable active participation in health care. The third one is *critical* health literacy and includes health-relevant decision-making, the provision of information health determinants, and opportunities to achieve change at the political and organizational level. Migrants often have lower levels of health literacy compared to native-born citizens due to health literacy requiring knowledge particular to the host society, such as who to ask for help, and what to expect (25). Therefore, health literacy stands out as a key variable for determining what access migrants have had to adequate healthcare during the pandemic. Health literacy is crucial and important for migrants to engage, react and act in relation to health issues that they face both individually and within communities in which they live.

The socio-ecological model (26) states that health is affected by close interaction between the individuals, the community and the environment, and includes social, physical and political components (27). This model describes five levels of influence: individual (e.g., skills, knowledge, attitudes), interpersonal (e.g., families, co-workers, social networks, friends), organizational (e.g., social institutions, organizations, schools, workplaces), the larger community (e.g., the relationship between organizations that work together), and public policy (e.g., national, national laws and regulations) (26). An individual's health sits at the center of the social nexus between these different levels.

Since refugees in Sweden are already facing social challenges with integration, living conditions and families facing separations through the migration process (6–9) as well as experiencing challenges in accessing health care (17), there is a need to further investigate how these issues have been handled during the pandemic. It is, therefore, important to prevent further suffering socially, physically, and mentally for refugees residing in Sweden. Recently published findings highlight the vulnerability of refugees during the COVID-19 period in relation to healthcare access and information as well as their living conditions (28). To help understand why refugees are particularly impacted by the pandemic, it is necessary to observe how health- and social workers have viewed refugees and their needs in relation to COVID-19. Importantly, such information does not negate the ongoing need to study the experiences of refugees themselves. In this study, however, we focus specifically on health- and social workers because they work closely with refugees. Therefore, the aim of this paper is to better understand how Swedish health- and social workers have reacted to the health- and social needs of refugees during the pandemic.

*Research questions:*

1. *What is the perception among health- and social workers on health care access and information for refugees in Sweden during the pandemic of COVID-19?*
2. *What have been the challenges and issues related to the social situation for refugees in Sweden during the pandemic of COVID-19?*

## Methodology

### Participants

The study focused on health- and social professionals that have worked with refugee adults or families during the recent pandemic of COVID-19. The inclusion criteria were the following: the participants should have been professionals within the health- and social sector such as nurses, social workers, midwives, head of health care clinics or pre-school teachers at the family health care centers. The mentioned health- and social professionals were chosen based on having regular contacts with refugees either within the health or social service in Sweden. Pre-school teachers were chosen since they work with open pre-school at family health centers and because they have regular contacts with refugee families. The open pre-school is a service within the family health care clinics, that offer open house gatherings for people living in those areas and they are staffed by pre-school teachers. Convenience sampling was implemented for recruiting informants. In total, 13 health- and social workers were interviewed in-depth, which was considered enough due to saturation of the data collected. The interviewees had on average 4.8 years of experience of working with refugees in either health- or social care settings. For information on the professions included, also considering the gender, please see Table 1.

**Table 1:** Number and type of health- and social workers interviewed.

**Profession**	**Number**	**Gender**	**Working experience with refugees**
Nurse	9	Female	Between 0.5 and 8 years, average 3.7 years
Midwife	1	Female	17 years
Social workers	2	Female	Between 6 and 8 years, average 7 years
Pre-school teacher	1	Female	7 years

### Materials and Procedure

The interview guide included questions on healthcare access before the pandemic as well as during the pandemic, and how information was communicated from the Public Health Agency and the Swedish healthcare system to the refugees. In addition, interviewees were asked to note their observations regarding what strategies they observed refugees used in order to protect their health, and how refugees adapted to a more digital health- and social care (see Appendix 1). Health- and social workers were also asked to describe what cultural knowledge they themselves used when working with refugees. The interview guide was based on earlier research (2–4), including qualitative interview studies (8, 29, 30). The interview guide was used in a semi-structured way, which means that the interviews were built on the questions in the interview guide but follow up questions were asked. The follow up questions were more open-ended, providing space for any necessary elaboration and clarification (31).

The first author accessed participants through contacts in Sweden that worked with refugees and asked if they or their colleagues were interested in participation in interviews. The contact network of health- and social workers that the first author reached out to, was built on earlier contacts through a current research project (32) using convenience sampling, where the first author reached out to all within her contact network that worked with refugees and that were willing to be interviewed. In total, nine interviews were conducted; seven of them were single interviews, and two of them were focus group interviews. There were both single interviews as well as focus group interviews, since it varied depending on interviewees' preferences. The interviews were done through zoom or through phone and lasted between 25 and 57 min. The interviews were conducted between December 2020 and June 2021.

### Analysis

The interviews were transcribed by the first author shortly after the interviews were conducted. The transcripts were read and coded by the first author in relation to the aim of this study. The coded material was read by the second and last author to ensure inter-rater reliability. The material was then analyzed following content analysis by Burnard et al. (33). The analysis was done manually, and categories and sub-categories were derived through manual coding on a word document where similar codes were grouped together and built into sub-categories, and then further grouped into categories. The analysis resulted in three main categories and seven sub-categories. Table 2 provides excerpts from the meaning unit, subcategory to category. The process of analysis was conducted as follows:

1. Interviews were read and transcribed verbatim.
2. Open coding was performed and used to create sub-categories.
3. After clarifying the meaning of each subcategory, they were grouped into categories.
4. The results were discussed by all three authors and adjustments were made continuously until all data were used (33).

**Table 2:** Examples from the analysis.

**Meaning unit**	**Code**	**Sub-category**	**Category**
It's the language that is the real problem and not the digital tools. I think that this group could manage the digital tool but when you have to understand it in another language, then that is the problem	It's the language and not the digital tools that is the barrier and the problem	Barriers for healthcare	Access to healthcare during a pandemic
During the spring and as long as we could remain open, we gave information to the refugees we met and we also put up posters with this information as well.	Giving information about COVID-19 and put up posters as well	To inform refugees about COVID-19	The need to share information about COVID-19
I have seen refugee families that experience more loneliness and isolation during the pandemic, and they feel like they don't have anywhere to go or any people to meet	Families are more isolated and lonely now	Social distancing for refugees and consequences of that	The need for strategies to stay healthy

## Results

The results were divided into three categories with associated sub-categories. The following categories emerged from the analysis: *Access to healthcare during a pandemic, The need to transfer information about COVID-19*, and *the need for strategies to stay healthy*. See Table 3, that shows the main categories and examples from each category. Table 4, shows how many meaning units we found per category and sub-category.

**Table 3:** Main categories and examples from each category.

**Main category**	**Examples from the interviews**
Access to healthcare during a pandemic	“*The child health care has a very good way and system of reaching even refugee families that are undocumented and through this way we reach almost all families here”* (Informant 4)
The need to share information about COVID-19	“*We taught Swedish for newly arrived refugee mothers and incorporated words related to COVID−19 in these classes in order to inform about COVID-19”*. (Informant 3)
The need for strategies to stay healthy	“*Along the pandemic and the struggles refugees had to face due to isolation, I have noticed that they suffer a lot and they drink more and take less care of themselves. And I am worried about this.”* (Informant 9)

**Table 4:** Number of meaning units per category and sub-category.

**Categories and sub-categories**	**Number of meaning units (percentage)**
**Access to healthcare during a pandemic**	131 (21.9%)
Reaching out to refugees in need of care	71 (11.8%)
Barriers for healthcare	60 (10.0%)
**The need to share information about COVID-19**	101 (16.9%)
To inform refugees about COVID-19	59 (9.9%)
Obtaining correct information on the right to healthcare	24 (4.0%)
**The use of translators for language brokering**	18 (3.0%)
The need for strategies to stay healthy	67 (11.2%)
Protecting refugees from COVID	33 (5.5%)
Social distancing for refugees and its consequences	34 (5.6%)

### Access to Healthcare During a Pandemic

The following category “Access to healthcare during the pandemic” resulted in two sub-categories: *Reaching out to refugees in need of care* and *Barriers for healthcare*. These sub-categories reveal how healthcare professionals reach refugees with healthcare both before and during the pandemic and what barriers have been witnessed during the pandemic.

#### Reaching Out to Refugees in Need of Care

According to the informants in the study, prior to the pandemic, refugee health clinics played a central role directing refugees to the healthcare system. These clinics are both financed by the regional governments as well as voluntary civil society. The refugee health clinics are serving refugees before they obtain refugee status and the social security number normally required for accessing services, as well as assisting them in finding health care centers and registering for support. Even before refugees receive refugee status, they have the right to receive healthcare that could not be deferred, meaning all healthcare that could not be postponed or that needed to be assessed the same day (34). Refugee health clinics help the refugees to navigate through legal obstacles when they consult, for example, the regular healthcare clinics and ensure that refugees get access to care that they have the right to access. The analysis of the interviews revealed that the refugee clinics follow up on referrals of care regarding refugees, in order to see that the refugees get the healthcare for which they have the right. For example, if the refugees have a certain physical ailment such as pain in the back or legs, for which they need to be investigated with x-ray and other certain consultations, the refugee health clinic follow them closely in order to see that they get the health assessments and consultations that they are in need of.

Child healthcare services, including school-nursing care settings, provide a primary entrance to healthcare, through which refugees access healthcare in Sweden: One nurse explained it like this:

“*There is a positive rumor about the child health care clinic in our area and many refugees come here and through that way we reach them.”* (Informant 4)

Further on she added:

“*The child health care has a very good way and system of reaching even refugee families that are undocumented and through this way we reach almost all families here.”* (Informant 4)

When the refugee families enter the child health care center, they can also get hold of all the other services that the family health care centers have such as maternity care, social care, and open pre-school. The child health care clinic has had success in reaching almost all refugee children in their areas and even refugee children that have not received permission to stay in the country. According to one of the interviewed nurses, the extended home-visit program, Grow Safely, which is a home-visit program where all first-time mothers receive six home-visits over a period of 15 months, with nurses, midwives, social workers and dentist assistants, has been effective in getting in contact with and supporting refugee families who were in need of early support (32, 35).

“*This program was effective in finding these refugee families through home-visits and the collaboration became better between the midwives and the nurses and in this way we can earlier find families in need of extra support”. (Informant 4)*

During the pandemic, the healthcare settings have remained open and have adjusted the reception areas with precautions and adjustments (36). The healthcare professionals consider that they have had the possibility of receiving patients in the same way as before the pandemic but have taken more contacts through phone than before. As pandemic restrictions increased, the social welfare workers were required to work from home and so supported other ways of keeping in contact with families in need. In response to the restrictions, the open pre-school for toddlers tried to engage with refugee families one-to-one to maintain contact with them. One-to-one contacts were arranged by the social workers and open pre-school that invited families to the family health care center at appointed time, allowing them to follow social distancing requirements whilst being able to meet the families in real life.

“*This was a good and safe way to meet one family at a time and through this way be able to keep contact and support the refugee families that are in need.” (Informant 3)*

#### Barriers for Healthcare

Healthcare professionals mentioned barriers to healthcare access for refugees, that existed prior to the pandemic, but became worse for refugees due to the restrictions. They witnessed refugees having difficulties reaching healthcare centers. Some refugees have been on waiting lists or referrals and are still waiting for appointments, challenging their already weak trust in the Swedish healthcare system. One nurse at the refugee health clinic said:

“*There are a few cases that have been waiting on referrals for healthcare and are still waiting for appointments. This is challenging since refugees already have a weak trust in healthcare settings and if you then are put on hold and wait, this could … reduce the trust in the Swedish society, which in the long-term, is never good.”* (Informant 2)

Further on, the same informant added regarding waiting times for appointments:

“*Some of the refugees worry that the long waiting time for referrals is an indication of their uncertain juridical status in Sweden.”* (Informant 2)

The interviewees also described situations when the healthcare centers were stopping patients at the entrance to check for symptoms of COVID-19. Some healthcare centers have stopped their drop-in service, which means that one cannot just enter without calling first, making it difficult for refugees not fluent in Swedish to access care, which is confirmed by recent research that shows that there has been a shift from face-to-face encounters within healthcare for refugees to more digital assessments and contacts (4, 37, 38).

“*This could be perceived as a barrier that is caused by a lack of Swedish knowledge among our migrants and therefore misunderstood and there was risk that they did not understand the purpose or the correct meaning behind the barriers that were put.”* (Informant 2)

Our interviews also showed that social services and open pre-school facilities at family healthcare centers were closed during the pandemic. Healthcare professionals, as a consequence, were anxious that they would lose contact with refugee families in need. One healthcare professional explained it like this:

“*It's the personal relationships that I am concerned with, that we now could lose, when we have closed the open preschool service in this area.”* (Informant 3) “*We are now afraid that we miss to support refugee families that are in special need of social support during the pandemic*.” (Informant 3)

For the interviewee, closing the open pre-school service, which is a part of the family health care clinics, made it harder to have *ad-hoc* meetings with refugee families. These spontaneous meetings with refugee families within the family health care clinics are important to catch up or be able to see refugee families that need extra support or help. This was confirmed by another healthcare professional, who saw that the lack of *ad-hoc* meetings also made it harder for them to engage with each other's clients, with the effect that the frequency of contacts between health and social care providers and refugees drastically reduced. This is confirmed by recent research that says that the pandemic has increased the challenges to collaborate between different professions and organizations (38). Engagements or close contacts between professions could for example be a midwife reporting to the child health care nurse that a refugee woman is soon giving birth, and that she would need extra support because she has been depressed during her pregnancy. These engagements were seen as often helping more families to get support at an early stage and before more serious things could occur and, as such, there was a fear that these issues were being ignored due to the pandemic restrictions. There could be specific needs related to living as a refugee, such as exposure to mental and physical health challenges that are related to economic stress and uncertainty over residency status. One nurse explained it like this:

“*I met a refugee woman that were feeling very bad mentally and needed help and support to get help with her mental health. I was able to support her and help her with access to health care regarding this matter.”* Informant 2

Another barrier for access to healthcare during the pandemic has been the use of digital services for testing and for booking appointments. The healthcare professionals highlighted that the obstacle was not the digital tool as a technical product, but rather its interface, that required both an advanced level of Swedish but also a Swedish bank ID account. One healthcare professional said:

“*The refugees cannot order the self-test for COVID-19 online without showing their identification through the Swedish bank ID. So, therefore, they seek care at the refugee health clinic, and we have to assist them with this service instead.”* (Informant 1)

The rapid shift to digital tools created new barriers for refugees not necessarily because of a lack of technical knowledge or even language barriers, but often first due to the inflexible demand that such tools require having a Swedish BankID—something often inaccessible to refugees in Sweden.

Health- and social workers also reported that while the public healthcare phone line usually has multiple language options (39), this function has been on hold during the pandemic due to priority of services, making it much harder for those not confident in explaining or expressing health issues in Swedish.

“*So instead, we as a refugee health clinic had to welcome and receive them here instead. For example, a refugee family needed health care since the woman in the family had much pain in her stomach and did not know where and how to seek care*.” (Informant 1)

### The Need to Share Information About COVID-19

The following category “The need to transfer information” resulted in three sub-categories: *to inform about COVID-19, to get the right information about the right to healthcare, and, to use translators*. These sub-categories reveal the importance of information spread during the pandemic but also how necessary it is for both refugees and health care professions, to have the correct information about the right for refugees to access health care. It also highlights the need to work with translators in order to transfer information correctly and avoid misunderstanding in communication and convey to the refugees their rights to access healthcare service.

#### To Inform Refugees About COVID-19

Several of the interviewees mentioned that they informed refugees in person but also through translated written information from the Public Health Agency (40). Although this information was translated, not all languages were available. One nurse explained that:

“*We realized that we did not have information translated into Somali, which was quite embarrassing since a lot of Somalis were hit by COVID in the area of Stockholm.”* (Informant 4)

A teacher from the open pre-school, which is a part of the family health care center and its service, expressed their work with information like this:

“*We taught Swedish for newly arrived refugee mothers and incorporated words related to COVID-19 in these classes in order to inform about COVID-19”*. (Informant 3)

The interviewees realized that some of the refugees had got information through social media and from home countries instead of through the Swedish Public Health Agency. They realized this from observing that refugees in the beginning were taking more precautions and kept children home from school, even if this was not recommended by the Swedish Public Health Agency. Paradoxically, it became clear that refugees were often trying to follow much stricter recommendations than those being communicated to them by Swedish health- and social workers, since they listened to the news from their home countries. When dealing with families that were taking what initially seemed excessive precautions, they tried to explain health in a broader way for these families:

“*Health is much broader than just the pandemic and keeping safe from that, for health is also to consider other aspects such as social factors and that children have a need and are obligated to attend school*.” (Informant 5)

The same healthcare professional at the Child health care center explained it also like this:

“*Instead of asking the families if they followed the recommendations, I asked how the family was doing and their situation at home”*. (Informant 5)

Sweden has been globally noted for taking a much more relaxed approach to the pandemic, at least at the start (41), relying more on voluntary self-restrictions over closing work places, for example. Within international media and political debates, Sweden has sometimes appeared as irresponsible for taking a more relaxed approach to the pandemic. In that context, refugees in Sweden following international social media could be seen as being more responsible, complicating their relationship with Swedish health- and social workers. One nurse expressed it like this:

“*They listen a lot to media in their home-countries in regard to information about precautions related to COVID-19 and we have felt that the refugee families either exaggerate or have a very relaxed approach to the media information.” (Informant 5)*

Healthcare workers and social workers in the current interviews reported that they faced challenges working with refugees due to the diverging guidance in foreign media, making it also difficult to maintain trust. This information was about precautions such as wearing face masks which in the beginning was not required in Sweden, but after a while started to be recommended. The shift in information between the home countries and Sweden's approach could make it difficult for refugees to know which information they should trust.

A midwife talked about the challenges with on-going and changing of information during the pandemic and how in the beginning the pregnant women were not a risk group and later on that changed:

“*In the beginning of the pandemic we told pregnant women to be careful, but they could still go out and were not considered to be a risk group, but later on the information changed and they were seen as an at- risk group and we had to adjust and withdraw the first information that was given*.” (Informant 8)

There were also ongoing changes in the information, such as if the partner could join the pregnant woman in maternity care facilities and if face masks should be worn. The midwives were afraid that the changing information and its differences with the foreign media refugees often relied on undermined refugees' trust in Swedish health guidance.

“*At the initial stages of the pandemic we informed the pregnant women that their partners could not join the check-ups at the maternity care but later on we informed them that they were allowed to bring their partners*.” (Informant 8)

#### Obtaining Correct Information on the Right to Healthcare

During the pandemic and as well prior to the pandemic, there has been a need to inform refugees about what kind of healthcare they have the right to obtain. There has been a need to clarify that even if refugees are undocumented, they still have the right to healthcare that could not be deferred (42). There has also been seen a need to clearly show and tell refugees that the health and social workers do not report any refugees to the police, although this is complicated by police having authority to access address lists held by social services.

The need for giving refugees the right information about the right to healthcare was required. One healthcare worker said it like this:

“*There have been healthcare staff that believe that refugees only have the right for emergency care and then have either forbidden people access to healthcare or given the healthcare under cover and without keeping it in the clinic‘s journal.”* (Informant 2)

The law on healthcare for refugees' requiring “healthcare that could not be deferred” (43) has caused a lot of misunderstandings, sometimes leading not to refugees getting the healthcare to which they are entitled. The law is actually more generous than is commonly understood, since healthcare that could not be deferred means that the refugees could and should get all the healthcare that could not wait until another day, which covers a wide range of conditions. The healthcare staff at one of the clinics working with undocumented migrants, have given priority to increase the knowledge and information about this law and the rights for refugees to obtain healthcare, no matter what legal right as refugees they have.

“*This information is very important since the law about healthcare rights is difficult to understand and could leave both refugees and healthcare workers with questions about its meaning*.” (Informant 2)

#### The Use of Translators for Language Brokering

Several healthcare professionals mentioned that the translation service has shifted from being on site to a phone service during the pandemic. There were complaints about the quality of translation phone services, including excessive background noises and even some driving whilst translating. Other complaints were from healthcare professions working with refugee children:

“*It was difficult during our visits with 4-year-old children and trying to make a language assessment while both having oral masks and at the same time using translators through phone and this makes it difficult for the child to hear and understand what to do and say during such a visit.”* (Informant 4)

The nurse was concerned with the risk that information/communication between the child, parents, nurse and translator is missing, which could deteriorate the ability to conduct correct language and development assessments for these refugee children.

Another comment came from a nurse describing it like this:

“*It is very difficult to help* refugee *women with breastfeeding assessment and support them and then you have to have translation through phone and not in the room where they are more able to translate according to what they see.”* (Informant 5)

Although there were some complaints about translation through phone, some of the healthcare professionals were satisfied with the translation and thought the healthcare work with refugees was carried on without language barriers.

### The Need for Strategies to Stay Healthy

#### Protecting Refugees From COVID

At the beginning of the pandemic, according to the informants, it was observed that there were more refugees wearing face masks compared to the rest of the population in Sweden. And, overall, the refugees were perceived to be more careful concerning social distancing by isolating themselves at home. They were also more likely to cancel health appointments and check-ups that they were supposed to attend with their infants. One nurse said:

“*In the beginning of the pandemic, the* refugee *families disappeared first and stayed home from visiting the open pre-school at the family healthcare center.”* (Informant 4)

There were also many refugee families that kept their children from attending pre-school, even if this was never recommended here. Several healthcare professions concluded that refugees had an avoidance strategy regarding social contacts, but later it also became apparent that the refugees have been more hesitant to seek vaccinations.

Healthcare workers noted their own confusion when trying to understand the divergent strategies taken by refugee families in response to the pandemic. A nurse interviewed said:

“*One family has totally self-isolated and goes only for visits to the child healthcare center, but then suddenly they plan to travel to Pakistan. I don't really understand it, but it must be some kind of desperate feeling of wanting to be close to family in spite of the risks*.” (Informant 5)

Often refugee families adopted restrictions earlier than the rest of the Swedish population, another nurse stating:

“*Refugees wore more masks and gloves at the beginning of the pandemic but now it feels weird since we have changed the recommendations during the year in Sweden, and have now lately been recommending precautions such as masks and gloves.”* (Informant 8)

Conflicting and changing understandings of what was needed regarding social distancing, have made it harder for healthcare workers and social workers to communicate with refugee populations, which is in line with recently identified challenges refugees face in obtaining information regarding COVID-19 (44). The Swedish case also showed that such refugees were not passive but, rather, often active in trying to protect their health during the pandemic by seeking alternative guidance that they saw as more trustworthy than that coming from Swedish public health authorities.

#### Social Distancing for Refugees and Its Consequences

Several of the nurses and social workers highlight the challenge where refugee families self-isolated by keeping themselves and their children home. They observed that early infants were kept home from pre-school and schools lost their Swedish language due to isolation. Another nurse reported other consequences of being more home:

“*We have noticed that our small children at the child health care have eaten more and done less physical activity and, therefore, gained weight since they were kept home and did not attend school or pre-school.”* (Informant 4)

The home environment was viewed by our interviewees as contributing to physical inactivity. The nurses at child health care also believed that if children were staying away from schools, it could slow down their learning process at schools, which could have serious consequences in the long-term and slow down their learning of the Swedish language.

Health- and social-workers reported that parents also complained to them that over-crowded living during the pandemic increased conflicts between their children. From the perspective of those interviewed, such family conflicts increased the risk of domestic violence.

A nurse working with undocumented refugees expressed her own concerns as follows:

“*Amongst the pandemic and the struggles refugees had to face due to isolation, I have noticed that they suffer a lot and they drink more and take less care of themselves. And I am worried about this.”* (Informant 9)

It is noteworthy that the general concern over the potential negative effects of isolation reflect those seen in other countries, but in Sweden health- and social-workers saw such views as particularly tied to refugees. This can be explained by the observation that refugees adopted more restrictive approaches to the pandemic compared to the rest of the Swedish population, marking them out as deviant. As such, what became common elsewhere stood out in Sweden as something mainly associated with those less likely to follow the guidance of the Swedish public health authority. A midwife saw that social distancing during the pandemic could be worse for newly arrived refugees due to their lacking a social network in Sweden. However, as noted, the Swedish health- and social-system was not focused on supporting refugees choosing to self-isolate, in part at least, because there was not officially the same demand on the population to self-isolate compared to other countries, according to the Public Health Agency of Sweden (45).

## Discussion

The aim of this study was to better understand how Swedish health- and social workers have reacted to the health- and social needs of refugees during the pandemic. The research questions were on perceptions of needs and challenges regarding health care access and the social situation for refugees in Sweden during the pandemic.

The interviews showed that during the pandemic, the healthcare settings have remained open and have adjusted the reception areas with precautions and changes in relation to COVID-19, as well as that the healthcare professionals consider that they have had the possibility of receiving patients in the same way as before the pandemic. But there have also been barriers for accessing health care which have been more evident than before the pandemic. The results of the study revealed that the translation service shifted from being on site to a phone service during the pandemic. There were complaints about the quality of translation and a risk that health information was lost in communication where translators' lack of physical presence meant that they were not able to follow the non-verbal communication in the rooms at the health service centers, which could be confirmed by recent research that shows that following non-verbal communication could be a challenge for refugees during encounters within healthcare settings (46). Furthermore, there were different ways that refugees chose to protect their own health, but that social distancing was challenging due to crowded living and being isolated, which was challenging because there was a lack of physical space in crowded apartments. Further on, many refugees were facing loneliness before the pandemic and the restriction with social distancing deteriorated this challenge. Prior to the pandemic, refugees were already exposed to unstable and crowded living conditions and suffered from lacking sufficient social contacts (6, 7, 9). These negative effects of the restrictions were perhaps made worse where refugees followed guidance based on a more restrictive approach than the one applied in Sweden, relying more on foreign media, and the Swedish health- and social services did not support these approaches until they only much later became recommended in Sweden (45), which is in line with the current study.

Regarding healthcare access, it is already known that refugees have faced barriers for accessing healthcare in Sweden (6, 7, 17). We also know that in times when health care access is generally restricted, refugees are worst affected since they already face significant barriers while seeking health care (3). This is as well-confirmed in a new-published report from the World Health Organization that also states that refugees during the pandemic have faced problems to access their healthcare settings (47). From the information obtained from the interviews, it was evident that healthcare and social workers continued to engage with the refugees and adjusted their work to keep reaching out with support and healthcare. But in spite of that, some barriers have been more evident than before, including the need to ensure effective communication with refugees (4, 37). This needs to be ensured for upcoming new challenges, for example with future pandemics that could arise and is very crucial since refugees already face both physical, mental and social challenges after arrival here and could be in great and urgent need of health and social care (6, 7). Recent research shows that there has been a shift from face to face encounters within healthcare for refugees to more digital assessments and contacts (4, 37, 38) and that is also confirmed in the new report from the World Health Organization (47). This could be necessary due to restrictions on social contacts but could be less efficient among refugees due to language challenges in the new country and lack of technical equipment to use digital tools such as health service apps. The need for proper communication is as well-highlighted by a new-published paper showing that refugees are at a risk of being excluded from the general information around COVID-19 in European countries (48). A new-published study from Sweden highlights the need for healthcare in Sweden to cover the gap between healthcare settings and refugee communities in Sweden and the authors suggest a more patient-centered care and a more culturally congruent health care environment (4). Furthermore, they see a need to empower refugees to participate in the health care decision-making process through engagement, which means close contact and collaboration between healthcare workers, decision makers and refugee communities such as religious leaders and trained health professional sharing the cultural background of minority groups. Through this engagement they could better understand the refugee communities and their cultural beliefs, values, language, context and/or behaviors of a specific cultural group (4) and through this contacts, knowledge gaps could be better covered for in the future. This needs to be ensured in order to reach the goal set in Sweden for equality in healthcare among all citizens in Sweden (4), which as our study indicates, has been shown to be lacking during the pandemic.

The results of the current study also revealed that healthcare and social workers were worried about the closure of the open pre-school service that is within family healthcare centers and especially important for vulnerable groups such as refugees that are in need of social relations and language practice. This needs to be considered when continuing the work with immigrants and be prepared for if future lock-downs could occur. There is a lack of socialization for many refugees, as well as language practice, especially for women that are having difficulties to enter the employment market (6, 7). The negative effects of COVID-19 on the integration of refugees is as well-mentioned by a recent study in Germany that highlights that there could be a setback in labor market integration for refugees due to the pandemic, which needs to be emphasized (49). Therefore, stakeholders in charge of health- and social care in Sweden as well as in other countries need to realize that such consequences could be faced both during and after the pandemic, which is in line with recent research about the situation for refugees in Sweden during the pandemic (4).

Furthermore, another challenge according to the interviews was the recommendation about social distancing. This group of refugees that the informants meet within their health- or social care are already facing challenges with crowded living conditions (9), which could deteriorate the situation for families being home more due to social distancing. Healthcare and social workers shared their doubts and concerns about refugee families having more conflicts during the pandemic which could become worse by overcrowded living accommodation (2, 4, 9). Social distancing also seemed to increase the feeling of isolation for refugees during the pandemic. The isolation could be felt by many during this period of time, but could be more negative for a vulnerable group such as refugees that both need fellowship as well as language practice. This is needed, in order not to prolong the period where they are not a part of the Swedish employment market, since difficulties with employment is common among newly arrived refugees in Sweden (7). According to the informants in the current study, the situation was also made worse by a lack of coherence between the relatively non-restrictive approach taken in Sweden with health guidance used by refugees often coming from outside Sweden where there was a wider consensus supporting a more restrictive approach. The challenge with different kind of media information through different channels, is highlighted in recent research, that concludes that “we are not only in a “pandemic” but also an “infodemic” (p. 676) (50). This epidemic of misinformation globally is spreading rapidly through social media platforms and other outlets and has had devastating consequences to public health and wellbeing. Further on, it increases the possibility for confusion in the pandemic, since different countries have implemented different mitigation measures (51). The differences in media information make finding reliable information even more problematic in regard to refugees' health literacy, when knowledge about COVID-19 is constantly changing, it is incomplete and depends on the context (51). The problem of misinformation is also far from simple given that, as in this Swedish case, refugees often proved well-informed on the need for pandemic restrictions given their use of international media and that meant they were initially ahead of the health- and social-workers. This needs to be considered when interpreting the results from the current interview study and it is crucial to ensure that refugees in Sweden have a good health literacy since it is required in order to resist the flow of information and to allow individuals to trust and be able to act upon reliable information and guidance (51).

The research questions focused on Swedish health- and social-workers' perceptions of needs and challenges regarding health care access and the social situation for refugees in Sweden during the pandemic. One aspect that was raised among the health- and social workers was the change in the content of the recommendations given. The recommendations were initially much less restrictive than internationally, changing only later on. For example, Sweden was late to introduce face masks or treat pregnant women as an at-risk group. The challenges that Swedish health- and social workers experienced when advising refugees who had access to health information that contradicted that provided by the Swedish public health authorities raises several interesting issues. It could be challenging since the Swedish health- and social workers are used to being authoritative when advising patients in general, and in the case of refugees acting contrarily and not follow the Swedish guidelines. The initial reaction we see in the interviews was that refugees choosing to, for example, isolate their families were marked as “deviants” by the health- and social workers. This could make it difficult for the health- and social workers in Sweden who rely on what the Swedish Public Health Agency says and closely follow that (45). But on the information obtained from the interviews, the severity of this label reduced over time and was, throughout, tempered by health- and social workers knowledge of the broader situation in which neighboring countries took much more restrictive measures than initially followed in Sweden. At the same time, it also highlights the extent to which refugees' own health literacy was undervalued whenever it deviated from the public health guidance coming from the Swedish public health authorities, and will little to no space for critical reflection among the health- and social workers working with refugees. The relationship between the Swedish health- and social workers with refugees appears to have been one-sided with health guidance communicated with little consideration of the receivers' existing health literacy (51). The lack of acknowledgment of their own knowledge coupled in a context of changing knowledge paradigms could be challenging for refugees that already have a low trust in institutions in society (6, 7) and could lead them to distrust the information and service that are given through health- and social care settings and could in a long-term deter them from adhering to the guidelines and information that are given. In turn, this risks making it harder for refugees to successfully utilize the Swedish healthcare and social services to support their health and life quality. The informants in the current study highlighted the extent to which refugees in society rely on an increasing diverse range of media and knowledge when seeking guidance on how to protect their health. Health literacy is therefore not only about having access to knowledge, but also understanding how to navigate the systems in which one is living, which is considered to be an essential aspect during COVID-19 in previous research (51). That may also require that health professionals and social workers in those systems are able to somehow engage with that diverse media. This aspect is important for health- and social workers to be aware of while working with refugees in the future, since refugees do not live in a vacuum, but their actions, individual and collective behaviors are influenced by changing environmental and personal factors and could be explained by the socioecological model (26). This model shows that there is a need for health literacy skills and considerations across all these levels of influence. Furthermore, it is clear that health literacy requires that health- and social-care systems are able to better communicate across those borders as diseases and treatment needs do not recognize national borders (51). The example of refugees' health- and social-support in Sweden during the pandemic illustrates this need.

## Conclusions

The current study revealed a few significant things. It showed that refugees are facing more barriers while seeking healthcare, as has been identified previously (17) but has been made particularly evident during the pandemic (47). Refugees in Sweden have in general taken precautions and followed recommendations but faced challenges with social distancing due to isolation and crowed living, which could affect their social integration and wellbeing in the long-term. Furthermore, it revealed that refugees used increasingly diverse sources of knowledge when trying to protect their health, and that not everyone had access to the knowledge needed to access healthcare and social systems. A recommendation from the current study is that efforts and improvements are needed in order to prevent ill-health for this specific group while facing similar challenges in the future. When interpreting the results of this study, health literacy is considered to be a crucial aspect in order for refugees to be able to understand, engage and react to changes (51) that happen during a pandemic such as the one that has been ongoing last years. However, health literacy is not a one-sided deficiency on the part of the individual, but needs to be understood as a negotiation between the individual and the healthcare system. This two-sided challenge needs to be further addressed to counteract further and future deficiencies. Future research is also needed regarding how the pandemic has affected migrants in the long-term in regard to how social distancing and closure of social gatherings have affected social integration among refugees in Sweden. But is also needed in regard to how healthcare access could be improved among refugees, especially since the healthcare have become more digital during the pandemic and could be assumed to be so as well in the future.

## Strengths and Limitations

We acknowledge several limitations within the current study presented here. In particular, there are limitations in relation to the recruitment of informants. The recruitment was made through contacts that the first author had with health- and social care settings in Sweden, utilizing a convenience sampling method. In addition, low number of informants recruited for the current study limits its capacity to provide a representative picture of the broader population. There was another limitation to the study, since no male professions were included as informants in the interviews, we were not able to reach a gender balance, which would have been relevant for getting perspectives from both genders. Since a convenience sampling was used, it was not possible to find any more informants than the ones included. Noting these limitations, we nonetheless argue that the chosen approach provides a useful case by which to enhance understanding of not only how Swedish health- and social-workers have treated refugees during the pandemic, but overall the complex challenges facing health equity.

The strength of the chosen recruitment process was that several different health- and social workers were recruited belonging to different healthcare settings, both governmental and private and voluntary networks. Although the sample of interviewees were collected both within governmental as well as private and voluntary settings, there was no purpose of exploring the differences between these settings, even if that could be of interest to compare. In addition, in relation to the limited data that has been collected, we are not able to see any differences between the different health- and social workers that were interviewed. But the fact that the health- and social workers were accessed from various settings could broaden the perspective of the opinions and attitudes among health- and social workers in Sweden. It could also be a disadvantage that a small number of health- and social workers from each profession were interviewed, which could limit the perspectives that were obtained through the interviews. Another negative part with the data collection in the current study, was that only one pre-school teacher was included in the interviews, but the reason behind the choice to do so, was that the pre-school teachers work very closely with the refugee families within the family health centers and their perspectives were important to include. However, since these interviewees work closely with each other in the different social and health settings, their perspectives were seen to complement each other in the interviews. A significance of the study is related to the transparent analysis process shown in Table 2 where the reader can see the process of the analysis related to this paper and that there is prior experience before in conducting several interview studies. Another strength of the method could be that the all analyzed data is presented including the number and percentage of how frequent each sub-category was mentioned in the data. It could also be discussed if the interview guide was able to catch the perspectives that the health- and social workers have on this matter or if it were too broad. The intention was to have quite open questions in the semi-structured interviews and let the interviewees tell from their perspectives. Follow-up questions were used in order to further discuss pertinent matters or perspectives that arose during the interviews. However, it is up to the reader to determine to what extent the results from this current study could be transferred to another setting or context (52).

## Data Availability Statement

The datasets presented in this article are not readily available because pursuant to national legislation, ethical review boards in Sweden do not allow release of sensitive raw data to the general public. Excerpts from the interviews have been translated and are a part of the results. Requests to access the datasets should be directed to elisabeth.mangrio@mau.se.

## Ethics Statement

Ethical review and approval was not required for the study on human participants in accordance with the local legislation and institutional requirements (53). The patients/participants provided their written informed consent to participate in this study.

## Author Contributions

EM carried out the interviews and analyzed the data. EM wrote the initial version of the manuscript and had detailed feedback from SZ and MS. All authors participated in the design of the study and have read and approved the final version of the manuscript.

## Funding

This research was funded by The Swedish Foundation for International Cooperation in Research and Education (STINT).

## Conflict of Interest

The authors declare that the research was conducted in the absence of any commercial or financial relationships that could be construed as a potential conflict of interest.

## Publisher's Note

All claims expressed in this article are solely those of the authors and do not necessarily represent those of their affiliated organizations, or those of the publisher, the editors and the reviewers. Any product that may be evaluated in this article, or claim that may be made by its manufacturer, is not guaranteed or endorsed by the publisher.
