# Supplementary material for: Working With Refugees' Health During COVID-19—The Experience of Health- and Social Care Workers in Sweden
Source: Front Public Health. 2022 May 19;10:811974. doi: 10.3389/fpubh.2022.811974 (PMC9163396; doi:10.3389/fpubh.2022.811974)
Supplement: Supplementary file 1 [file Data_Sheet_1.PDF]

1. How well has the health and social work functioned in relation to before the pandemic? If it has not been functioning well, have you been able to reach the refugees with health- and social care?
2. How have you informed refugees of public health agency recommendations?
3. How would you describe the social situation for refugees during the pandemic?
4. How has the health situation been for refugees during this period? What are the common symptoms of ill-health that you have observed?
5. What strategies have you noticed that the refugees have shown in order to protect their health?
6. How would you describe health care access for refugees during the pandemic (were the refugees able to receive health and social care when needed)?
7. How has it been working for refugees to adapt to a more digital contact with health care and social sector? Have the refugees been able to participate and access care through this way?
8. How has the translation service worked during the pandemic?
9. What do you, as a professional, think that we could learn from refugees during the recent pandemic?
10. What intercultural competence training is needed?
